# Double Opposing Perpendicular Linear Repair of Gauge Ear-Piercing Deformity: A New Technique and Literature Review

**Published:** 2014-09-05

**Authors:** Matthew R. Zeiderman, Shahrooz Sean Kelishadi, John Paul Tutela, Saeed Chowdry, Bradon J. Wilhelmi

**Affiliations:** Division of Plastic and Reconstructive Surgery, University of Louisville School of Medicine, Louisville, Ky

**Keywords:** gauge ear piercing, earlobe repair, pierced ear deformity, ear reconstruction, dilated earlobe

## Abstract

**Objective:** The repair of dilated ear lobules after gauge ear piercing is increasingly performed to restore the natural ear appearance and shape for esthetic, professional, or social reasons. Because of a deficit of remaining lobule tissue, reconstruction of this area can be challenging. Many have described the repair of partial cleft ear lobule defects, but few focus on the repair of dilated ear lobules. The authors review the methods of repair described in the literature and propose a new technique. **Methods:** A double opposing perpendicular linear closure was used to repair dilated ear lobules. A linear closure is made on the anterior aspect of the circular lobular defect followed by a linear closure on the posterior aspect oriented 90 degrees to that of the anterior surface. **Results:** This method produces an esthetically pleasing result with a rounded, natural appearance. Perpendicular repair lines prevent the dog-ear deformity that may be noticed on the lobule with a single linear closure. Preserving the outer remnant of ear lobule prevents notching seen when this area is violated. **Conclusions:** Several different techniques have been described for repair of the dilated ear lobule that has been deformed by gauge ear piercing. We describe a new method that is simple to perform and successful in restoring the dilated ear lobule.

In the United States, approximately 34% of people aged 18 to 50 years have pierced ears.^1^ In 2013, more than 40,000 patients received cosmetic ear surgery,^2^ including a segment seeking correction of partial or complete clefts of the earlobe due to trauma or heavy earrings. Gauge ear piercing has become increasingly popular among young adults in recent decades. This technique involves piercing of the ear with gradual dilation and stretching of the ear lobule after wearing plugs of increasing diameter. As a result, an increasing number of patients seek esthetic reconstruction for deformities of the dilated ear lobule when gauge ear piercing is no longer desired. The increased prevalence of gauge ear piercing and eventual correction likely contributes to the 81% increase in cosmetic ear surgery from 1997 to 2013.^2^

Unlike the linear clefts seen in patients with “traditional earrings,” large gauge piercing creates a circular defect frequently larger than 5mm, leaving only a small portion of the inferior rim of the lobule intact. Consequently, the increased demand for correction of dilated ear lobule deformity presents a challenge for reconstructive plastic surgeons that is unique from the repair of the linear cleft ear deformity.

After gauge ear piercing, the resulting deformity creates a lobule stretched beyond its normal boundaries in diameter while at the same time lacking normal tissue in between; the majority of the prepiercing lobule architecture is either missing or thinned out. Many different techniques have been proposed for the correction of torn earlobes resulting in either complete or partial clefts, but few focus on the repair of dilated ear lobules after gauge ear piercing.^3^^-^5 Here, the authors describe a new technique for repair of the dilated ear lobule after gauge ear piercing.

## METHODS

**Figure 1:**
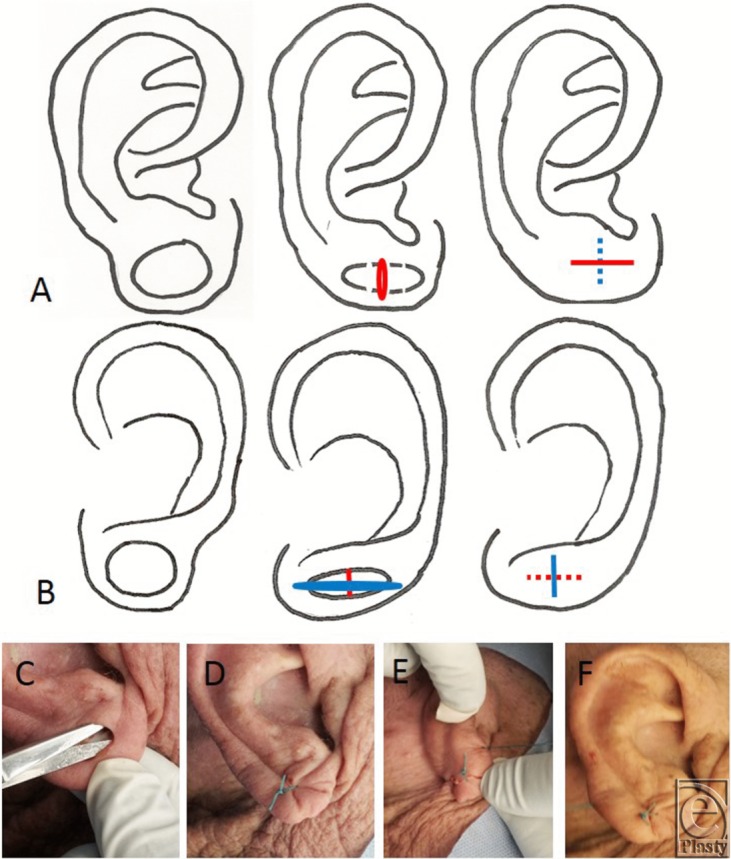
(*a*) Drawing of repair method, anterior aspect of ear. (*b*) Drawing of repair method, posterior aspect of ear. Intraoperative views of (*c*) scoring the defect, (*d*) 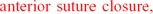 (*e*) 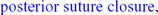 and (*f*) completed procedure.

We used a double opposing perpendicular linear repair. This procedure can be performed using local anesthetic and with 5-0 nylon sutures. A graphical depiction of this method is demonstrated in Figures 1a and 1b to complement this description. The procedure is initiated by scoring a circle within the circular rim of the defect. The scored circle is then undermined to fat as shown in Figure 1c. A linear closure is then placed on the anterior aspect of the circular lobular defect (Figs 1a and 1d) followed by a linear closure on the posterior aspect oriented 90 degrees to that on the anterior surface (Figs 1b and 1e). A single linear reapproximation of tissue anteriorly and posteriorly in the same direction would distort the lobule and also result in a dog-ear deformity. Performing a posterior closure perpendicular to the anterior closure corrects the medial and lateral dog-ear deformity resulting from the anterior approximation. Two perpendicularly opposed linear closures pull the ear lobule together in a way such that it is rounded, free of notching, appears natural, and is esthetically pleasing with minimal scarring and is demonstrated in Figure 1f.

## RESULTS

**Figure 2:**
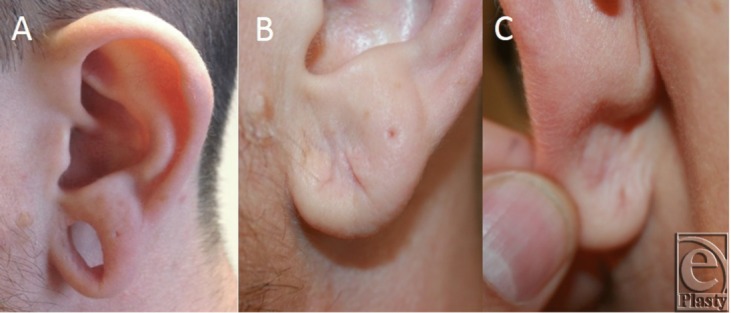
(*a*) Preoperative view of a patient with 25-mm diameter gauge piercing for 14 years. One year postoperative photographs of the (*b*) anterior aspect and (*c*) posterior aspect of the ear lobule.

Figure 2a represents the average ear lobule deformity seen after gauge ear piercing. Figures 2b and 2c reveal representative postoperative results at 1 year using this method to produce a rounded, esthetic ear lobule. Six patients had successful reconstruction of ear lobule defects after gauge ear piercing using the double opposing perpendicular linear closure technique described for defects measuring 10 mm to 25 mm. At 1 year postoperative evaluation, all patients had satisfactory outcome as determined by patient and physician satisfaction with the esthetic appearance of the repaired lobule.

## DISCUSSION

In 1954, McLaren^6^ was the first to document repair of earlobe clefts using a simple linear closure under general anesthesia. Since then, many others have gone on to describe a variety of different techniques for the repair of partial cleft earlobe deformities due to elongation of the piercing, often secondary to gravity or trauma. Tan^7^ described the punch technique for earlobe clefts smaller than 4 mm, which utilizes a punch biopsy perpendicular to the earlobe to de-epithelialize the defect with subsequent straight-line closure and healing. Abenavoli^8^ reported the closure of partial cleft earlobe deformity using a half Z-plasty technique with a good esthetic outcome for 10 patients. Miller and Eisbach^9^ have reported closure of small dilations of the ear lobule by conversion of a partial cleft to a complete cleft through the inferior rim of the lobule with subsequent repair via Z-plasty.

An innovative approach for repair of partial clefts was proposed by Reiter and Alford,^10^ who described a method using a “parallel opposed flap”; this technique creates a flap on the anterior medial surface and the posterior lateral surface, pulling each flap through the cleft and suturing the flaps at the raw exposed areas. This technique is suitable for smaller defects but less reliable for large dilations of the lobule where less local tissue is available to create flaps.

Vujevich and Obagi^11^ described a purse-string method for the closure of partial cleft defects. This method excises the epithelium of the partial cleft and passes a suture in a purse-string fashion through the inner margin of the cleft to draw the lobule together. This method has many uses but may be less precise when applied to larger defects; larger defects usually have asymmetry with much thinner, narrower remnant lobule at the inferior rim compared to the rest of the remaining tissue. Also, this technique relies on 1 continuous suture tied to itself bearing all of the inherent tension of the wound.

The aforementioned methods described the repair of partial clefts but do not focus on the repair of dilated earlobes as a result of gauge ear piercing. While they may be suitable methods of repair for partial clefts, their applicability to dilated lobule defects is limited. The repair of the dilated earlobe is a distinct defect, as it is not amenable to linear closure if a round, esthetic result is to be attained. This is because of significant stretching and thinning of the tissue of the inferior rim of the ear lobule. The literature contains scattered reports of ways to repair the gauge ear deformity, many of which are brief. In the ideal repair, reconstruction of the dilated ear lobule will yield a rounded, natural appearing esthetic result, is amenable to future piercing, is easily reproducible, and displays minimal scarring. The historical evolution of repair techniques for dilated ear lobule defects from gauge ear piercing are discussed and how they satisfy these objectives.

**Figure 3:**
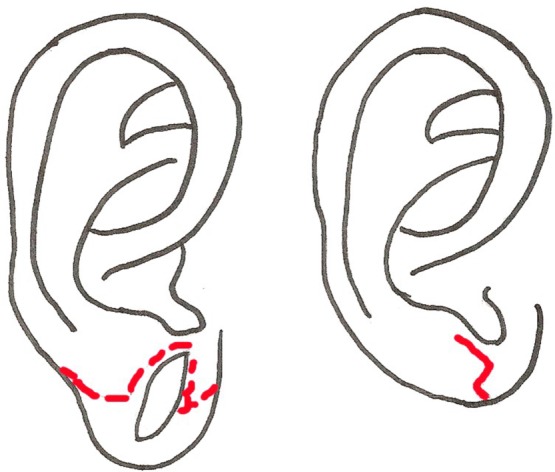
Method described by Williams and Majumder.

Williams and Majumder^12^ proposed removal of the inferior portion of the dilated earlobe tissue and repair of the hole by de-epithelialization of the margin and subsequent approximation of the remaining earlobe (Fig 3). They describe this method of repair in 2 patients. Their results state significant reduction of the dilated lobule, creating an earlobe that is slightly smaller and flatter than it was prior to gauge ear piercing and is amenable to repiercing. Because this method requires removal of tissue from a defect that already lacks adequate tissue, imprecise cuts could lead to suboptimal reconstruction and less ideal secondary options for reconstruction. Therefore, careful planning and precise execution of this technique is required.

**Figure 4:**
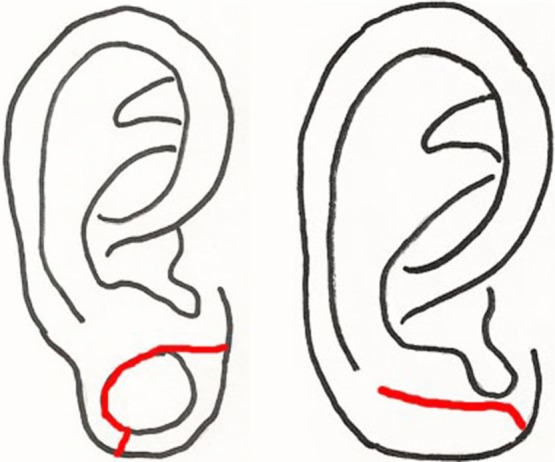
Method described by Henderson.

Henderson and Malata^13^ described a similar approach for repair of dilated ear lobules. They removed the excess tissue from the anterior half of the defect, and the remaining posterior flap of tissue is subsequently de-epithelialized and sutured to the tissue of the superior rim of the lobule in an L-shaped pattern (Fig 4). This method has been used for 6 ears on 3 patients and produces a good esthetic result. A potential limitation of this approach is that sufficient tissue must be present on both the superior and inferior rims of the ear lobule for reapproximation without tearing of the tissue, and to provide adequate bulk to yield a rounded, natural appearance. Insufficient tissue may lead to a small lobule with a flatter, less rounded appearance. The lobule will be slightly smaller than its original size due to tissue removal, so both ears must be repaired precisely in order to produce a symmetric result. Like many other techniques, this method has difficulty restoring symmetry in patients with unilateral defects.

**Figure 5:**
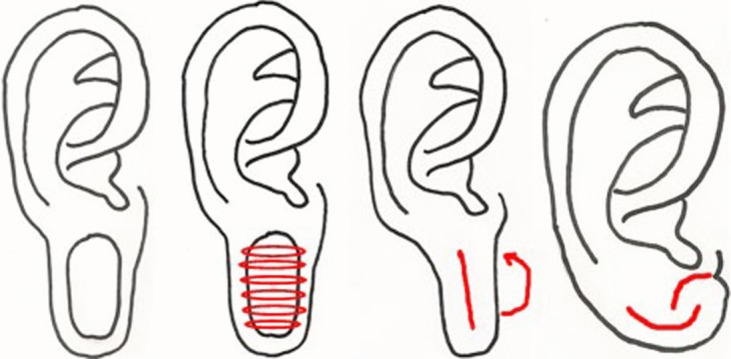
Method described by Arasatnam and colleagues.

Using a different approach, Arasaratnam and colleagues^14^ proposed approximating the dilated earlobe linearly and then folding the elongated tissue anteriorly to provide bulk for reconstruction (Fig 5). They reported satisfactory long-term outcomes with an unspecified number of patients. This technique preserves the tissue of the rim of the lobule, avoiding further destruction to the earlobe. It is useful for patients with larger dilations where there is little tissue remaining on both the superior and inferior rims of the gauge ear piercing. Adequate bulk is restored to the lobule, and there is a reduced risk of recurrent clefts with repiercing.^14^ However, the long-term esthetic result is unknown based on photos published in the literature.

**Figure 6:**
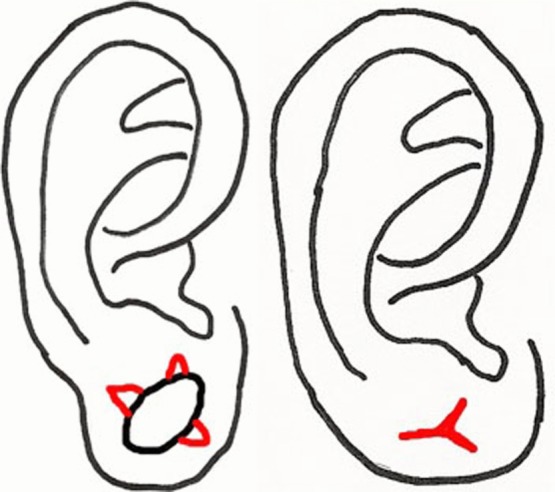
Method described by De la Sotta and colleagues.

De la Sotta et al^15^ described a procedure which creates 3 wedge excisions around the rim of the dilated lobule and then approximates the wedges with sutures on the anterior and posterior surfaces of the lobule (Fig 6). This procedure restores earlobe length and shape while maintaining the natural rim of the lobule without notching and has thus far produced a good esthetic outcome for a small number of patients.^15^ This method may provide adequate repair for many patients and requires only minimal tissue excision, but it does require sufficiently thick tissue on the rims of the lobule to make reliable interdigitating triangular flaps. As such, patients with thin inferior rims of the lobule may not be ideal candidates for this procedure.

**Figure 7:**
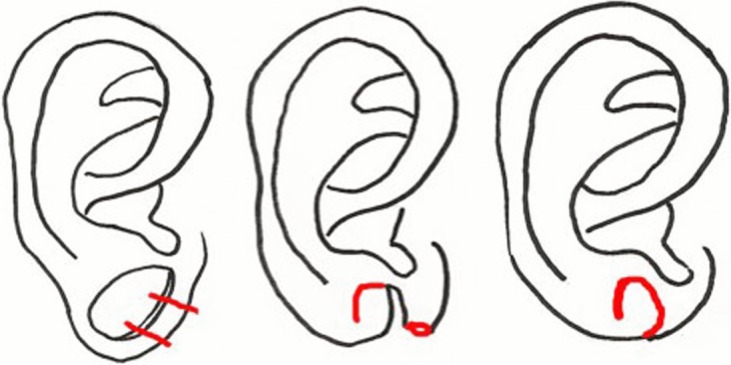
Method described by Bastazini and Pianaro.

Bastazini and Pianaro proposed a method of repair that folds the inferior rim of the dilated lobule superiorly and avoids tissue removal to recover the original volume of the lobule (Fig 7).^16^ An incision is made near the insertion of the anterior aspect of the enlarged lobule and the resulting flap is subsequently de-epithelialized. The flap is folded vertically upon itself and any excess tissue is removed. The former outer surface of the lobule is de-epithelialized and sutured to the remaining tissue near the anterior insertion of the lobule. This technique seeks to restore the lobule to the original volume and can be used for both small and large dilations. This method is a good option for patients with unilateral gauge piercing ear deformities in achieving better ear lobule symmetry. However, this technique requires more meticulous steps than other methods and there is an increased risk of flap necrosis because it relies on a narrow pedicle to attain the natural shape of the lobule.^16^ Less robust blood supply to the resulting flaps may lead to undesirable long-term esthetic results such as pin-cushioning. A symmetrical appearance of the earlobes can be achieved with this method, but the long-term esthetic result is still in question due to the significant area for scar tissue formation.

**Figure 8:**
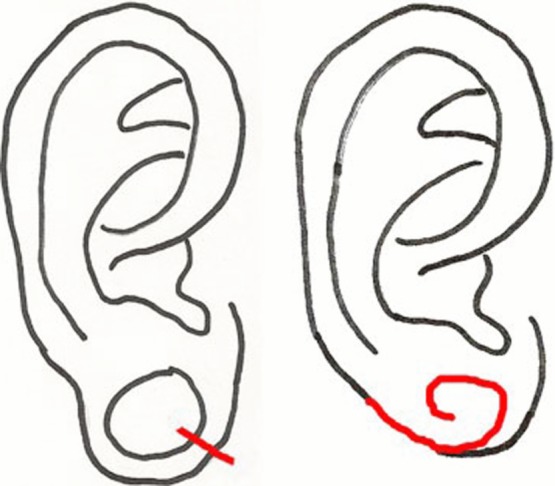
Method described by Snell and Caplash.

Finally, Snell and Caplash^17^ described a “rolled-up anchovy” method (Fig 8). The dilated lobule is first severed at the junction of the anterior first and second third of the tissue ring. The posterior surface of the anterior flap and both surfaces of the longer posterior flap are subsequently de-epithelialized and the posterior flap is rolled upon itself like an anchovy and secured by the shorter anterior flap, which serves as a cover. This method provides a full earlobe with more vertical height, and the diameter of the lobe can be tailored to the patient's preference. In addition, this procedure is particularly suitable for patients with abnormally large gauge ear piercing but may be more difficult to perform for less extensive gauge ear piercing due to the requirement of adequate flap surfaces for rolling. Like the method by Bastazini and Pianaro, the repaired lobule relies on a narrow pedicle, which increases the risk of pedicle failure and necrosis when healthy issue is scarce. However, when performed correctly, this method creates an acute angle at the root of the lobule and yields a fuller lobe and more vertical height than other methods.^17^

Repair of the dilated ear lobule with the double opposing perpendicular linear closure adds to the armamentarium of techniques described. Its biggest strength is that it is easy to perform and reproduce. With 2 linear closures oriented 90 degrees to each other, in addition to producing an esthetically appealing rounded appearance, the dog-ear deformity common to simple linear closures is avoided. When repairing an ear lobule that is compromised with thinning or deficiency of healthy tissue, this less complex procedure allows preservation and incorporation of valuable remaining tissue. Furthermore, unlike other methods, this procedure does not violate the thin inferior rim of the lobule, thereby minimizing scar tissue and subsequent risk of tear. Also, a lack of scar traversing the outer rim of remaining ear lobule prevents notching with contraction that occurs as a result of wound healing. As such, this method is ideal for larger defects for which other methods may yield an unappealing result or require violation of the inferior rim of the lobule (Figs 2a–2c). Another added advantage of this method is that it does not rely on flaps based on narrow pedicles and thus has less risk of necrosis and failure. In situations where little remains of the ear lobule, any tissue loss is devastating and makes salvage procedures less likely.

As patients with gauge ear piercing continue to seek repair of their dilated ear lobules, the described methods will be further refined and new methods will be described. We believe our proposed method to be a simpler yet powerful procedure for the reconstructive plastic surgeon when repairing the dilated ear lobule after gauge ear piercing.
